# Prevalence of Gastrostomy-Related Procedures in Polish Children: A Longitudinal Nationwide Analysis

**DOI:** 10.3390/jcm14196727

**Published:** 2025-09-24

**Authors:** Karolina Wyszomirska, Adam Wyszomirski, Michał Brzeziński, Anna Borkowska, Agnieszka Zagierska, Maciej Zagierski, Agnieszka Szlagatys-Sidorkiewicz

**Affiliations:** 1 Department of Pediatrics, Gastroenterology, Allergology and Nutrition, Faculty of Medicine, Medical University of Gdańsk, Nowe Ogrody 1–6, 80-803 Gdańsk, Poland; michal.brzezinski@gumed.edu.pl (M.B.); andzia@gumed.edu.pl (A.B.); agnieszka.zagierska@gumed.edu.pl (A.Z.); maciej.zagierski@gumed.edu.pl (M.Z.); agnieszka.szlagatys-sidorkiewicz@gumed.edu.pl (A.S.-S.); 2 Department of Adult Neurology, Faculty of Medicine, Medical University of Gdańsk, M. Skłodowskiej-Curie 3a, 80-210 Gdańsk, Poland; adam.wyszomirski@gumed.edu.pl

**Keywords:** gastrostomy, children, endoscopic, enteral feeding, nutrition

## Abstract

**Objectives:** This study aimed to determine the prevalence of gastrostomy-related procedures and paediatric patients undergoing these procedures, alongside temporal trends, patient profiles, and regional disparities in procedure provision. **Methods**: We analysed anonymised National Health Fund data pertaining to patients who underwent procedures at regional centres in Poland from 2010 to 2019. To assess temporal patterns, procedure counts and patient prevalence were aggregated annually, and trends over time were evaluated using the Mann–Kendall trend test. **Results:** Percutaneous Endoscopic Gastrostomy (PEG) was performed in 2638 patients, while gastrostomy-other was performed in 2087 patients. The cumulative prevalence of patients during the 10-year follow-up period was as follows: PEG: 37.7 per 100,000 children; gastrostomy—other: 29.9 per 100,000 children. The procedure prevalence rates were PEG: 40.1 per 100,000 children; gastrostomy-other: 43.0 per 100,000 children. Gastrostomy-related procedures were performed most frequently in the first three years of life. **Conclusions:** Patient sex, age, and centre location influenced utilization, as shown by local variations. The increasing trend in gastrostomy procedures and the young patient age indicate the growing use of gastrostomy, with endoscopic placement being the most common. Given the rising numbers requiring gastrostomy, optimizing inter-centre collaboration could contribute to the identification and treatment of patients with special nutritional needs.

## 1. Introduction

Children are a group of patients characterised by dynamic growth and development, which translates to high nutritional needs. Dysphagia and malabsorption are associated with many chronic diseases affecting paediatric patients and can lead to malnutrition and the need for specialised treatment. Long-term enteral access, such as gastrostomy, is an important element of this treatment if dietary supplementation is required for longer than 4–6 weeks.

Endoscopic or surgical gastrostomy enables nutritional treatment be provided while preserving the physiological pathway of the distal gastrointestinal tract in patients in whom optimal oral nutrition has become impossible. Obtaining this alternative access is relatively safe [1,2,3], and its continued use is effective, as shown by the improvement of nutrition levels among patients [4,5,6]. The use of artificial access into the stomach has been studied both qualitatively, by assessing the effectiveness of different gastrostomy procedures (endoscopic or surgical) [1,7,8,9,10,11,12,13,14,15,16] and the quality of patients and caregivers’ life following gastrostomy creation [3,6,17,18,19,20,21], and quantitatively, by analysing the incidence of complications [1,2,4,7,17,22,23,24,25] and changes in anthropometric indices [4,17,22].

Although gastrostomy placement is a commonly used procedure, data on the frequency, prevalence, and trends in the use of gastrostomies and on the profile of patients receiving these procedures nationwide remain limited. The prevalence of gastrostomy insertion has so far been studied within a narrower range of data, such as the prevalence of a first gastrostomy placement [4,16,26,27,28,29]; in populations of patients with Down’s syndrome [30], congenital anomalies [31], intellectual disabilities [32], and cerebral palsy [33]; and with regard to single-centre experiences [22,27]. Few papers address the prevalence of gastrostomy in large paediatric populations [34,35]. In view of the increasing prevalence of the Home Enteral Nutrition (HEN) procedure in children in many European countries [36,37,38], including Poland [39,40], an analysis of the epidemiology of gastrostomies remains important for further planning and reliable patient care.⁠ The basis of nutritional treatment, an important element of which is the creation of a gastrostomy, remains the effective identification of patients who require this specialised treatment.

In Poland, all gastrostomy procedures are performed in paediatric patients in hospital centres, which report data on the procedures performed to the National Health Fund according to the ICD—9 (The International Classification of Diseases, Ninth Revision). An analysis of data obtained from the National Health Fund, which is the sole payer for these procedures, allows for the assessment of the epidemiology of all gastrostomy-related procedures performed in children. The data were completely representative of nationwide prevalence.

The aim of this study was to determine the prevalence of endoscopic gastrostomy placement and procedures related to the surgical creation of access or replacement of a gastrostomy tube and the prevalence of paediatric patients undergoing these procedures in Poland between 2010 and 2019, as well as the assessment of temporal trends, patient profile (patients’ age, sex, and principal diagnoses), and regional differences in the provision of these procedures using nationwide register data.

## 2. Materials and Methods

The study is a retrospective analysis of gastrostomy tube insertion or gastrostomy tube replacement procedures performed in children in Poland between 2010 and 2019. We analysed anonymised data obtained from the National Health Fund—the only public health service, free of charge in Poland—relating to all paediatric patients who underwent procedures at regional centres between 1 January 2010 and 31 December 2019. Procedures were coded according to the International Classification of Diseases, Ninth Revision (ICD—9), and the assessment covered procedures with the following codes:

(a) Code 43.11 (including extensions): percutaneous endoscopic gastrostomy—this procedure includes endoscopic gastrostomy, hereafter described as PEG;
(b) Code 43.19: other gastrostomy—this procedure includes surgical (laparoscopic or open) gastrostomy creation and procedures for replacement of the primary or subsequent gastrostomy tube, further described as gastrostomy-other;
(c) Code 44.321: percutaneous endoscopic gastrojejunostomy—in this procedure, PEG-PEJ tubes are inserted, hereafter described as PEG-PEJ.

Data on the number of patients and the procedures provided to them between 2010 and 2019 were obtained, broken down by the individual ICD—9 procedures described above, as reported on 22 November 2022.

Epidemiological information on age, sex, geographic distribution, and principal diagnoses (consistent with the data as of 27 May 2022) was obtained without division into the individual ICD—9 procedures discussed. These data were combined for all ICD—9 procedures (sum for 43.11, 43.19, and 44.321 procedures), described further as “gastrostomy-related procedures”.

The number of patients aged 0–18 years who underwent one of the procedures between 2010 and 2019 and the number of procedures performed on them in this area were described.

The data on the total number of patients and procedures and the data relating to the profile of patients come from two separate reports obtained from the National Health Fund and show numerical differences due to a change in the state of the database.

These differences referred mainly to the number of procedures and did not exceed 19 annually. Additionally, one patient could have undergone PEG and gastrostomy-other procedures in a given year and thus be included in the number and prevalence of both analysed procedures.

Epidemiological data also had minor inconsistencies in the numbers, related to the fact that the considered patient’s age was the age at the time the service was provided. Therefore, if a patient received two services in a given year, one before their birthday and the other after it, they were counted twice in age ranges and prevalence by age group (double-counting affected a maximum of 7 patients annually). However, the extent of the differences in numerical values did not significantly affect the quality of the data and the conclusions of this study.

The cumulative and annual prevalence of paediatric patients undergoing PEG and gastrostomy-other and of these procedures in the paediatric population in Poland between 2010 and 2019 was investigated.

In the further stages of the study, prevalence was analysed taking into account children’s sex and age at the time the procedure was provided (including replacements).

Territorial differences in the prevalence of patients and procedures performed in different voivodeships of Poland were assessed. In 2010, 2011, 2013, 2015, and 2018, within a single voivodeship (Zachodniopomorkie, Lubelskie Świętokrzyskie, or Opolskie), the number of patients and/or procedures was <5; for calculation purposes, a numerical value of 4 was assumed for these data.

The principal diagnoses described according to the International Statistical Classification of Diseases and Health Problems (ICD—10) and reported to the National Health Fund at the time the procedures were performed were analysed.

Categorical data were presented as counts and percentages. Statistical analyses were performed, and figures were generated using the statistical software R, version 3.6.3. The investigation of the total number and prevalence of patients undergoing procedures (excluding prevalence by sex) was extended by developing time trends; data were aggregated by year, and the Mann–Kendall trend test was applied. A *p*-value < 0.05 was considered statistically significant.

## 3. Results

Between 1 January 2010 and 31 December 2019, PEG was performed in 2638 patients aged 0–18 years, who were provided with 2803 procedures, while gastrostomy-other was performed in 2087 patients, provided with 3003 procedures.

The report data obtained from the National Health Fund for PEG-PEJ was limited, as only for 2012, 2014, 2015, and 2019 were the actual number of patients (24 in total) and the procedures provided to them (44 in total) presented; for the rest of the period, these numbers were reported as a value <5.

Table 1 presents the number and prevalence of patients undergoing procedures and the number and prevalence of the procedures.

The results of this study show a statistically significant upward trend in the number of patients in successive years of analysis.

The prevalence of patients in the Polish paediatric population combined during the 10-year follow-up period was (a) PEG = 37.7/100,000 children; (b) gastrostomy-other = 29.9/100,000 children. The prevalence of procedures was (a) PEG = 40.1/100,000 children; (b) gastrostomy–other = 43.0/100,000 children.

The prevalence of patients and procedures by age group and sex in the Polish paediatric population combined during the 10-year follow-up period is shown in Table 2.

Gastrostomy-related procedures were performed in children of both sexes most frequently in the first three years of their life; it was found that in this age group, the prevalence of patients and performed procedures was the highest.

Most of the procedures in the entire analysed period were performed in Wielkopolskie Voivodeship (1325 services, prevalence of 197.03/100,000 children), and the highest prevalence of patients was recorded in the Warmińsko-Mazurskie Voivodeship (93.3/100,000 children); while in the analysed period, the highest number of patients provided with the discussed services was in the Mazowieckie Voivodeship (720, compared to 253 children in Warmińsko-Mazurskie Voivodeship). The voivodeship with the lowest number of patients and procedures was the Opole region (patients = 68, procedures = 73), while the lowest prevalence of patients and procedures was recorded in the Lubelskie Voivodeship (patients = 34.18/100,000 children, procedures = 37.01/100,000 children).

Patients’ principal diagnoses reported to the National Health Fund at the time of the procedures are shown in Table 3.

Throughout the analysed period, the most frequently reported principal diagnosis was attention to artificial openings, with the percentage of this diagnosis decreasing in successive years of follow-up. It accounted for 23.2% of all diagnoses in 2010 vs. 12.9% in 2019. In 2019, the leading diagnosis was symptoms and signs concerning food and fluid intake; the reporting frequency for this diagnosis increased over time (from 2.3% in 2010 to 17.3% in 2019).

In the described period, the most common groups of diagnoses were R—Symptoms, signs, and abnormal clinical and laboratory findings (23.4%); Z—factors influencing health status and contact with health services (17.4%); and K—diseases of the digestive system (14.2%).

## 4. Discussion

All public medical centres that performed gastrostomy procedures in children reported their services to the regional branches of the National Health Fund. The rules for settlement remain uniform for all centres, and that made it possible to obtain data on all paediatric patients who underwent insertion or surgical replacement of a gastrostomy in Poland between 2010 and 2019. Thus, this study provides the first description of the prevalence of gastrostomy procedures in the paediatric population in Poland over a long-term follow-up period. To the best of the authors’ knowledge, the analysis presented here also represents the only study presenting the prevalence of patients undergoing gastrostomy procedures in the entire paediatric population with an extensive description of this prevalence.

The study shows a statistically significant upward trend in the number of patients in successive years of follow-up (Table 1) and indicates the development of gastrostomy use in Polish children as well as among children of Western Australia, and other European countries [26,27,28].

PEG was the leading procedure in terms of the number of patients undergoing it, and this method appears to be the preferred method of gastrostomy tube placement. The total number of gastrostomy-other procedures and the average number of these procedures per patient were higher than those of PEG. However, the fewer total number of patients undergoing gastrostomy-other procedures, probably indicates that successive procedures (including gastrostomy tube replacement) are performed in the same children rather than surgery being the first choice of method for gastrostomy creation. This thesis is supported by the prevalence of endoscopic gastrostomy noted by other investigators [17,26,27,28,29,32,35]. Moreover, according to other researchers’ opinions and the authors’ own practice, the more common PEG use presented in this study could be attributable to clinical preferences [26] and the use of official ESPGHAN guidelines [41]. The frequency of the PEG-PEJ procedure was the lowest, which is consistent with other studies [29,36].

During the 10-year follow-up, a differentiated prevalence of patients and procedures among Polish children was observed (Table 1).

Pardy et al. indicate a prevalence of 83.7/100,000 children for gastrostomy in 2020; however, this study concerned only some children of the London population [29]. In Poland, a year earlier, the prevalence of gastrostomy procedures (total of PEG and gastrostomy-other) was 9.17/100,000 children, which was significantly lower than in the Swedish paediatric population (16.3/100,000 in 2019) [35]. Between 2010 and 2014, the prevalence of gastrostomies in the Dutch population was estimated at 50–55/100,000 children/year [34]; in the corresponding period in Poland, it was lower, ranging between 4.84 and 7.92/100,000 children/year. The prevalence of the procedures in the Australian paediatric population was 67/100,000 births, but this value concerned first-time gastrostomy and covered a longer follow-up period [26].

The inability to perform clinically meaningful comparative analyses on the prevalence of gastrostomy procedures in different countries draws attention to the need for uniform, population-wide studies in order to obtain the information necessary to optimise European guidelines for nutritional patient care.

Over a 10-year follow-up period, a variable prevalence of procedures in different age groups was observed (Table 2), with the highest prevalence in the group of patients under three years of age. A similar age structure was described by other researchers [4,14,27,34], and a decreasing trend in the age of children undergoing gastrostomy was shown [23].

A study on the prevalence of gastrostomy in the Polish adult population indicated its increasing number of patients in all age groups, excluding the youngest adults of 18–24 years of age [42]. Our study shows (Mann–Kendall trend test, Table 2) no increase in rates of patients undergoing gastrostomy-related procedures only in the adolescent group (14–18 years of age). This highlights the need for careful management of patients during the transition from paediatric to adult care. On the other hand, despite the lack of an upward trend, the relatively large group of adolescents observed in the current study may indicate effective nutritional intervention at the paediatric care stage. This could partially explain the low rates of young adults receiving gastrostomy procedures after the age of 18 years. Glasson et al. indicated an increasing trend only in younger age groups [26].

Procedures were performed more frequently in boys (54.8%), with the prevalence of patients and procedures in each age group being higher for males (Table 2). Numerous studies on various aspects of gastrostomy also described a predominance of male patients [12,14,26,27,28,32,33]. The reported male predominance is partly explained by the higher prevalence of central nervous system disorders in boys, which represent the most common principal diagnosis in children on HEN [23,36,40].

**Figure 1:**
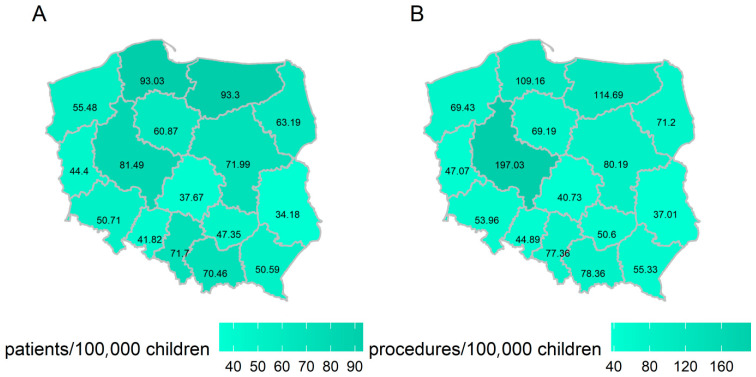
The prevalence of patients (**A**) and procedures (**B**) in the population of children in the relevant provinces of Poland between 2010 and 2019.

An uneven prevalence of patients and procedures in different regions of Poland was observed (Figure 1). Given this uneven prevalence and the previously described [39] territorial differences in the prevalence of paediatric patients who qualify for the HEN procedure as well as of nutritional centres in Poland, the authors again draw attention to the possibility of difficulty in accessing nutritional care for patients in some regions of the country. Optimizing patient care when specialistic medical centres are located at a greater distance should be based on efficient resource management, effective communication, and coordination between medical centres, implementing best practices from high-efficiency centres [43].

Healthcare administrators responsible for nutritional care in paediatric patients should support collaboration between paediatric departments and centres specializing in endoscopic procedures and enteral nutrition. Furthermore, education about the indications for gastrostomy and standards of care for enteral nutrition (recommended by scientific societies) could improve the specialist treatment of patients with nutritional needs.

The nature of the data reported to the National Health Fund does not allow an unambiguous identification of the most common principal diagnoses in patients undergoing procedures. Usually, the reported analysis procedure does not specify either the indication or disease of a patient. However, taking into account the most common ICD—10 group of diagnoses reported, these data allow us to indirectly infer the main indications for gastrostomy in children in Poland, being dysphagia (11.1%) and feeding difficulties (10.8%).

The described principal diagnoses (Table 3) partly coincide with the most common indications for gastrostomy reported by other researchers [1,7,17,24,29]. In our analysis, neurological disorders representing diagnoses most commonly resulting in a gastrostomy corresponded to only 3.9% of the diagnoses identified.

The European Society for Paediatric Gastroenterology, Hepatology and Nutrition (ESPGHAN) emphasises the need for the indications for gastrostomy to be considered on an individual basis by the interdisciplinary team [23]. A standardised system for reporting the indications and diagnoses of patients undergoing gastrostomy could prove helpful in further effective identification of patients requiring enteral feeding.⁠

## 5. Conclusions

The present study showed some potentially significant findings on the epidemiology of gastrostomy-related procedures in children in Poland. The use of gastrostomy-related procedures is influenced by the patient’s sex and age as well as the location of centres performing procedures in children, as indicated by the local differences described.

The upward trend in the performance of PEG and gastrostomy-other procedures during the 10-year analysis period and the young age of patients eligible for the procedures indicate dynamic development in the use of gastrostomy tubes in children in Poland.

The territorial differences in the prevalence of patients and gastrostomy-related procedures shown in the study are associated with a risk of impeded access to nutritional care for some patients. With the increasing number of children undergoing gastrostomy procedures, some of whom may have problems with receiving the required care, it is important to optimise inter-centre collaboration to effectively identify and successively treat patients with special nutritional needs.

## Figures and Tables

**Table 1:** Annual number and prevalence of paediatric patients and procedures for PEG and gastrostomy-other per 100,000 children in Poland, 2010–2019.

		PEG ^1^		Gastrostomy-Other	
Year	Paediatric Population (*n*)	Patients (Prevalence)	Procedures (Prevalence)	Patients (Prevalence)	Procedures (Prevalence)
2010	7,140,156	191 (2.68)	202 (2.83)	160 (2.24)	187 (2.62)
2011	7,146,551	188 (2.63)	202 (2.83)	158 (2.21)	171 (2.39)
2012	7,066,768	252 (3.57)	269 (3.81)	191 (2.7)	257 (3.64)
2013	6,995,362	254 (3.63)	274 (3.92)	180 (2.57)	277 (3.96)
2014	6,942,996	332 (4.78)	355 (5.11)	218 (3.14)	330 (4.75)
2015	6,901,795	298 (4.32)	313 (4.54)	222 (3.22)	303 (4.39)
2016	6,895,878	268 (3.89)	284 (4.12)	239 (3.47)	361 (5.24)
2017	6,920,652	258 (3.73)	276 (3.99)	285 (4.12)	451 (6.52)
2018	6,935,523	257 (3.71)	271 (3.91)	221 (3.19)	386 (5.57)
2019	6,948,706	340 (4.89)	357 (5.14)	213 (3.07)	280 (4.03)
2010–2019	Mann–Kendall trend test	tau = 0.51, *p*-value = 0.049		tau = 0.56, *p*-value = 0.032	

^1^ PEG, Percutaneous Endoscopic Gastrostomy, *p*-value < 0.05 was considered statistically significant.

**Table 2:** Age- and sex-specific number and prevalence (per 100,000) of patients and procedures for gastrostomy-related procedures among children in Poland, 2010–2019.

Age Group	Paediatric Population (*n*)	Patients (Prevalence)	Procedures (Prevalence)
	Total/Girls/Boys	Total/Girls/Boys	Total/Girls/Boys
0–2	1,164,679/566,045/598,634	1506 (129.31) ^#^/687 (121.37)/819 (136.81)	1845 (158.41)/815 (143.98)/1030 (172.06)
3–5	1,188,571/578,309/610,262	673 (56.62) ^#^/312 (53.95)/361 (59.15)	904 (76.06)/405 (70.03)/499 (81.77)
6–9	1,563,289/761,120/802,169	787 (50.34) ^#^/371 (48.47)/416 (51.86)	1032 (66.01)/495 (65.04)/537 (66.94)
10–13	1,506,999/734,184/772,815	718 (47.64) ^#^/322 (43.86)/396 (51.24)	867 (57.53)/420 (57.21)/447 (57.84)
14–18	1,565,900/763,540/802,360	836 (53.39) ^#^/352 (46.1)/484 (60.32)	1093 (69.8)/477 (62.47)/616 (76.77)

# Mann–Kendall trend test: Age group 0–2, tau = 0.73, *p*-value = 0.004; Age group 3–5, tau = 0.52, *p*-value = 0.047; Age group 6–9, tau = 0.60, *p*-value = 0.020; Age group 10–13, tau = 0.58, *p*-value = 0.025; Age group 14–18, tau = 0.20, *p*-value = 0.474; *p*-value < 0.05 was considered statistically significant.

**Table 3:** Distribution of primary diagnoses among paediatric patients undergoing gastrostomy-related procedures with number and percentage of cases.

ICD—10 ^1^ Diagnoses	Number of Patients	%
Other *	1125	23.70%
**R Symptoms, signs, and abnormal clinical and laboratory findings, not elsewhere classified**	1117	23.40%
R13 Dysphagia	529	11.10%
R63 Symptoms and signs concerning food and fluid intake	515	10.80%
R62 Lack of expected normal physiological development	73	1.50%
**Z Factors influencing health status and contact with health services**	826	17.40%
Z43 Attention to artificial openings	816	17.20%
Z51 Other medical care	5	0.10%
Z93 Artificial opening status	5	0.10%
**K Diseases of the digestive system**	677	14.20%
K21 Gastro-oesophageal reflux disease	375	7.90%
K31 Other diseases of stomach and duodenum	176	3.70%
K22 Other diseases of oesophagus	63	1.30%
K29 Gastritis and duodenitis	49	1.00%
K92 Other diseases of digestive system	14	0.30%
**J Diseases of the respiratory system**	415	8.80%
J96 Respiratory failure, not elsewhere classified	330	7.00%
J18 Pneumonia, organism unspecified	39	0.80%
J15 Bacterial pneumonia, not elsewhere classified	28	0.60%
J95 Postprocedural respiratory disorders, not elsewhere classified	13	0.30%
J20 Acute bronchitis	5	0.10%
**E Endocrine, nutritional, and metabolic diseases**	284	5.90%
E43 Unspecified severe protein-energy malnutrition	63	1.30%
E41 Nutritional marasmus	56	1.20%
E46 Unspecified protein-energy malnutrition	53	1.10%
E84 Cystic fibrosis	47	1.00%
E44 Protein-energy malnutrition of moderate and mild degree	43	0.90%
E88 Other metabolic disorders	11	0.20%
E71 Disorders of branched-chain amino-acid metabolism and fatty-acid metabolism	6	0.10%
E76 Disorders of glycosaminoglycan metabolism	5	0.10%
**G Diseases of the nervous system**	184	3.90%
G80 Cerebral palsy	96	2.00%
G40 Epilepsy	41	0.90%
G12 Spinal muscular atrophy and related syndromes	29	0.60%
G93 Other disorders of brain	13	0.30%
G41 Status epilepticus	5	0.10%
**Q Congenital malformations, deformations, and chromosomal abnormalities**	88	1.90%
Q39 Congenital malformations of oesophagus	65	1.40%
Q40 Other congenital malformations of upper alimentary tract	12	0.30%
Q04 Other congenital malformations of brain	6	0.10%
Q87 Other specified congenital malformation syndromes affecting multiple systems	5	0.10%
**S Injury, poisoning, and certain other consequences of external causes**	17	0.40%
S06 Intracranial injury	17	0.40%
**P Certain conditions originating in the perinatal period**	10	0.20%
P07 Disorders related to short gestation and low birth weight, not elsewhere classified	10	0.20%
**I Diseases of the circulatory system**	5	0.10%
I46 Cardiac arrest	5	0.10%

* Diagnoses that affected fewer than 5 patients are included in the “other” category; percentages may not add up to 100% due to rounding. ^1^ ICD—10, The International Classification of Diseases, Tenth Revision.

## Data Availability

The raw data supporting the conclusions of this article will be made available by the authors on request.

## Abbreviations

The following abbreviations are used in this manuscript:

PEG	Percutaneous Endoscopic Gastrostomy
HEN	Home Enteral Nutrition
ICD—9	International Classification of Diseases, Ninth Revision
PEJ	Percutaneous Endoscopic Jejunostomy
ICD—10	International Statistical Classification of Diseases and Related Health Problems, Tenth Revision
ESPGHAN	European Society for Paediatric Gastroenterology, Hepatology and Nutrition
